# Pulmonary Involvement in Recurrent Respiratory Papillomatosis: A Systematic Review

**DOI:** 10.3390/idr16020016

**Published:** 2024-02-28

**Authors:** Illari Sechi, Narcisa Muresu, Biagio Di Lorenzo, Laura Saderi, Mariangela Puci, Stefano Aliberti, Ivana Maida, Michele Mondoni, Andrea Piana, Giovanni Sotgiu

**Affiliations:** 1 Department of Medicine, Surgery and Pharmacy, University of Sassari, 07100 Sassari, Italy; illarisechi@yahoo.it (I.S.); imaida@uniss.it (I.M.); piana@uniss.it (A.P.); 2 Department of Humanities and Social Sciences, University of Sassari, 07100 Sassari, Italy; nmuresu@uniss.it; 3 Clinical Epidemiology and Medical Statistics Unit, Department of Medical, Surgical and Experimental Medicine, University of Sassari, 07100 Sassari, Italy; dilorbiagio@gmail.com (B.D.L.); mvpuci@uniss.it (M.P.); 4 Department of Biomedical Sciences, Humanitas University, 20072 Pieve Emanuele, Italy; stefano.aliberti@hunimed.eu; 5 Respiratory Unit, Department of Health Sciences, ASST Santi Paolo e Carlo, University of Milan, 20122 Milan, Italy; michelemondoni@gmail.com

**Keywords:** recurrent respiratory papillomatosis, HPV, lung cancer, lung involvement, pulmonary involvement

## Abstract

Recurrent respiratory papillomatosis (RRP) is a non-malignant disease, characterized by the production of wart-like growths in the respiratory tract, affecting both young people and adults (juvenile-onset recurrent respiratory papillomatosis, JORRP, and adult-onset recurrent respiratory papillomatosis, AORRP, respectively). Infection caused by human papillomavirus (HPV) is known as the main factor involved in RRP development. Complications of RRP may rarely occur, including lung involvement and malignant transformation. The present systematic review aimed to evaluate the prevalence of severe complications, such as lung involvement and lung tumor in JORRP and AORRP patients, and assess the role of HPV genotypes in the progression of disease severity following the guideline for reporting systematic reviews and meta-analysis (PRISMA Statement). A total of 378 studies were found on PubMed and Scopus using the following MESH terms: “recurrent respiratory papillomatosis and lung tumor” and “pulmonary tumor and recurrent respiratory papillomatosis”. Basing on inclusion and exclusion criteria, a total of 11 studies were included in the systematic review. We found a pooled prevalence of 8% (95% CI: 4–14%; I^2^: 87.5%) for lung involvement in RRP patients. In addition, we found a pooled risk difference of 5% in lung involvement between JORRP and AORRP (95% CI: −7–18%; I^2^: 85.6%, *p*-value: 0.41). Among patients with lung involvement, we observed a pooled prevalence of lung tumor of 4% (95% CI:1–7%; I^2^: 67.1%) and a pooled prevalence mortality for this group of 4% (95% CI:2–6%; I^2^: 0%). Overall, the positivity rate for HPV-6 and -11 in patients with RRP was 91%. Considering only cases with pulmonary involvement, the pooled prevalence for HPV-11 was 21% (95% CI: 5–45%; I^2^: 77.2%). Our results evidenced a low/middle risk of pulmonary involvement and lung tumor in JORRP and AORRP patients, with an increased risk for HPV-11-positive patients. Further studies should be performed to improve knowledge and adopt preventive measures to contrast the progression to severe diseases in RRP patients.

## 1. Introduction

Recurrent respiratory papillomatosis (RRP) is a benign clinical condition of the respiratory tract associated with an infection caused by human papillomavirus (HPV), especially HPV-6 and -11. While the majority of the cases are associated with low-risk HPV genotypes, a small fraction (less than 5%) is sustained by HPV-16 and other high-risk HPV genotypes [1]. RRP is a relatively rare disease, which can affect both children (juvenile-onset, JORRP) and adults (adult-onset, AORRP) with an approximately estimated incidence of 4 per 100,000 children and 2 per 100,000 adults [2,3,4,5]. However, a relevant decreased incidence of RRP was observed following the implementation of vaccination campaigns, as described in Australia and the US [6]. It is characterized by the development of multiple papillomas in the connective tissue of the upper respiratory tract, with a particular predilection for the larynx [2]: lesions, varying in size and showing rapid growth rates, can favor voice alterations, persistent cough, and airway blockage. Respiratory symptoms can significantly impact the quality of life, affecting breathing, speaking, and overall daily functioning [2].

In adulthood, RRP can occur in individuals aged 20–40 years, whereas JORRP usually develops in children 1–4 years old, without any significant differences in incidence based on sex, race, or ethnicity [7,8,9,10]. Transmission pathways of juvenile-onset and adult-onset forms are likely distinct. Previous studies showed that adult onset, following sexual transmission, is more prevalent in individuals practicing oral sex and having multiple partners [1,5]. Conversely, JORRP is attributed to exposure to genital warts during pregnancy or delivery: a maternal history of condylomas was associated with a 200-fold increased risk, along with a higher risk during vaginal delivery in comparison with Cesarean delivery [5]. The most frequent clinical symptom in children is hoarseness, whereas dysphonia dyspnea, dysphagia, upper respiratory tract infections, and pneumonia are the most common conditions in adults [2,3,5]. However, the natural course and severity of diseases can vary according to individual and virological characteristics. Being diagnosed at a young age is the most significant factor related to disease severity and progression. In fact, a diagnosis in children aged <3 years results in a risk almost four times higher of undergoing repeat surgery and complications, following the involvement of other anatomical sites [2]. The ability of the virus to replicate latently in epithelial cells is the main cause of recurrence, whereas an efficient immune response can prolong the periods of remission [2]. 

Prevention is based on HPV vaccination, and surgery is the first therapeutic option, followed by adjuvant therapies (e.g., interferon-α2a, antivirals) to reduce the high risk of relapse [11,12]. Laser excision represents the most common treatment, whereas tracheotomy may become necessary in cases of more severe disease [5]. However, scientific evidence did not recommend a frequent administration of adjuvant therapy because of its adverse effects, which include the risk of carcinogenicity and teratogenicity [5]. Moreover, the routine distribution of quadrivalent and nonavalent HPV vaccines (Gardasil^®^), which can protect against HPV-6, -11, -16, -18, -31, -33, -45, -52, and -58, can decrease the incidence of RRP preventing HPV infection. They can play a therapeutic role in reducing the number of recurrences in patients treated with standard surgical therapy [11]. 

Although the anatomical sites infected by HPV are mainly larynx and trachea, malignant transformation in bronchi and lungs (<1% of all lung neoplasms) can rarely occur, representing the primary cause of death in this patient population [9,13,14,15,16]. Malignancy, which can occur after surgical/chemotherapeutic therapies or after a relapse, within decades from disease onset [17,18,19], is the consequence of translocation of fragments from the larynx to the lung tissue after therapeutic manipulations [20,21]. Moreover, several studies suggest the potential role of other individual (i.e., age, immune response) and virological (i.e., HPV genotype, integration of the viral DNA into host genome) variables involved in the development of lesions, including those in the lower airway respiratory tract [8,10,18]. However, scanty evidence on the risk factors implicated in lung/pulmonary involvement and dysplasia in RRP is currently available [8,10,18]. 

A systematic review was carried out in order to assess the prevalence of complications, including pulmonary involvement and lung tumor and the mortality in RRP. Moreover, the secondary aim was to assess the prevalence of HPV infection and of its genotypes to evaluate their role in the development and progression of pulmonary lesions.

## 2. Materials and Methods

### 2.1. Search Strategy

The protocol of the present systematic review was recorded on PROSPERO (registration ID CRD42023426064). Articles focused on the prevalence of pulmonary disease and mortality in patients with RRP published on PubMed and Scopus databases were retrieved. The following strings were chosen for database search: “recurrent respiratory papillomatosis and lung tumor” and “pulmonary tumor and recurrent respiratory papillomatosis”. All studies published between 1 January 1997 and 31 December 2022 were evaluated. Reference lists of previously published reviews and selected studies were also assessed to include articles excluded by the search engines. 

### 2.2. Study Selection

All observational retrospective and prospective studies describing pulmonary involvement in RRP patients were included. Exclusion criteria were articles written not in English language, commentaries, letters, reviews, and case-reports or -series with fewer than 10 patients.

After removal of duplicates, records were independently screened by two authors (I.S. and B.D.). Following the assessment of titles and abstracts, full texts were carefully evaluated and suitable articles were selected to be included in the systematic review. If no consensus could be reached, a third author (G.S.) was consulted to resolve the conflict. 

### 2.3. Data Extraction

The following study characteristics and outcomes were extracted: first author and title of the article; year of publication; year/s when the study was conducted; follow-up; study design; country/ies where the study was performed; demographics (i.e., sex and age); sample size; lung involvement or pulmonary lesions in JORPP and AORRP; mortality; lung tumor; type of lung involvement, HPV prevalence and HPV genotypes. 

### 2.4. Study Quality Assessment 

The inter-rater agreement for study selection and data extraction was ~100%, and only a few inconsistencies were solved by consensus and support of a third investigator (G.S.). Guidelines of the Preferred Reporting Items for Systematic Reviews and Meta-Analysis (PRISMA) were adopted [22], as well as the Scottish Intercollegiate Guidelines Network [23] and the Joanna Briggs Institute Critical Appraisal tools (JBI) [24] to assess the quality of the observational and experimental studies, respectively.

### 2.5. Statistical Analysis

Study characteristics were summarized with descriptive statistics: qualitative variables with absolute and relative (percentage) frequencies and quantitative variables with means and standard deviations (SD) or medians and interquartile range (IQR), respectively. 

Forest plots were used to represent study variability with 95% confidence intervals (CI) for the following outcomes: prevalence of lung involvement in all samples and stratified by HPV-11 infection, lung tumor, and mortality. Heterogeneity was measured with the inconsistency indicator (I^2^), where an I^2^ value > 50% indicated substantial heterogeneity. Fixed- or random-effects models were chosen taking into consideration the expected between-study heterogeneity. Egger weighted regression test methods and bias assessment plots were used to assess publication bias. A two-tailed *p*-value less than 0.05 was considered statistically significant. The statistical software StatsDirect version 3.1.12 (StatsDirect Ltd., London, UK) and STATA version 17 (StatsCorp, College Station, TX, USA) were used.

## 3. Results

### 3.1. Study Selection

**Figure 1:**
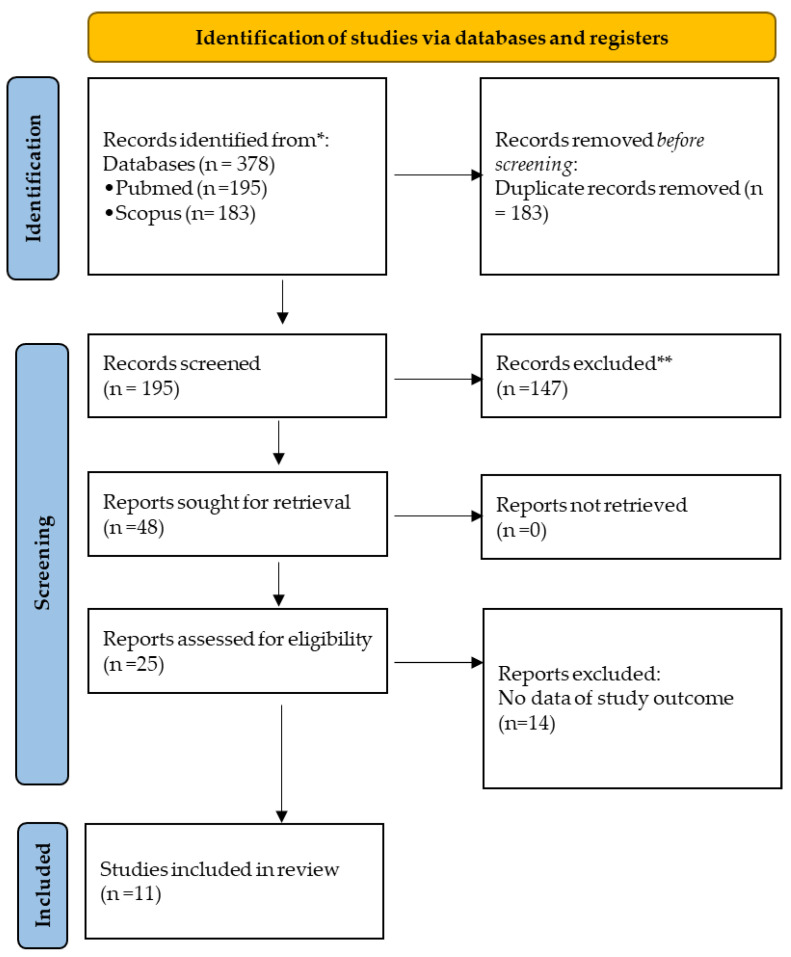
PRISMA 2020 flow diagram for new systematic reviews that included searches of databases and registers only. * Consider, if feasible to do so, reporting the number of records identified from each database or register searched (rather than the total number across all databases/registers). ** If automation tools were used, indicate how many records were excluded by a human and how many were excluded by automation tools.

Overall, 378 records were initially found. After removal of duplicates (183; 48.4), 147 (38.8) articles were excluded, as they were deemed unrelated to the primary and secondary aims. Subsequently, a total of 48 studies were screened by titles and abstracts; 23 (48%) of those were excluded, as they did not report outcomes of the study or they were reviews or cases-series with <10 subjects. A total of 25 full texts were evaluated, and 11 articles were selected (i.e., 44% of the records screened at the initial phase of the process) (Figure 1).

### 3.2. Quality Assessment

The majority of the studies (10; 90.9%) had a retrospective/observational study design. The quality assessment showed a high risk of bias in the majority of the studies (8/10, 80%) [25,26,27,28,29,30,31,32,33], whereas only two had a moderate risk (2/10, 20%) [34,35] (Table 1). 

Only one with an “experimental-phase II” design [31] was classified as high risk of bias (Table 2). 

### 3.3. Characteristics of the Selected Studies

Studies were published between 1997 [30] and 2021 [25,34]. The recruitment period ranged from 1981 [30] to 2020 [25]. Study designs were mainly retrospective (7/11, 63.6%) [26,27,30,31,32,34,35], followed by prospective/longitudinal (2/11, 18.1%) [28,29] and experimental (1/11, 9.0%) designs [31]; only one article did not report the design of the study (1/11, 9.0%) [25]. A total of eight (72.7%) studies [26,27,29,30,31,32,33,34] were monocenter, two (18.2%) [28,35] multicenter, and one (9%) [25] did not clarify the number of clinical centers involved in the research. Studies were mainly conducted in the US (5, 45.4%) [25,29,30,31,33], followed by China (1.9%) [34], Norway (1.9%) [31], Russia (1.9%) [27], Germany (1.9%) [28], Australia (1.9%) [30], and Poland (1.9%) [35] (Table 1, Table 3 and Table S1).

### 3.4. Characteristics of the Study Samples

The sample size ranged from 12 [31] to 448 [27] patients, for a total of 1382 patients. Among the 1278 subjects included in the analysis, a total of 950 (74.3%) [27,29,30,32,33,34] and 290 (22.7%) [25,26,31,35] were classified as JORRP (1–18 years) and AORRP (>18 years), respectively. Only one study did not discriminate between JORRP and AORRP, for a total of 38 patients [28]. Information on gender was reported by 11 (100%) studies, with a total of 731 males and 535 females. 

### 3.5. Outcomes

**Figure 2:**
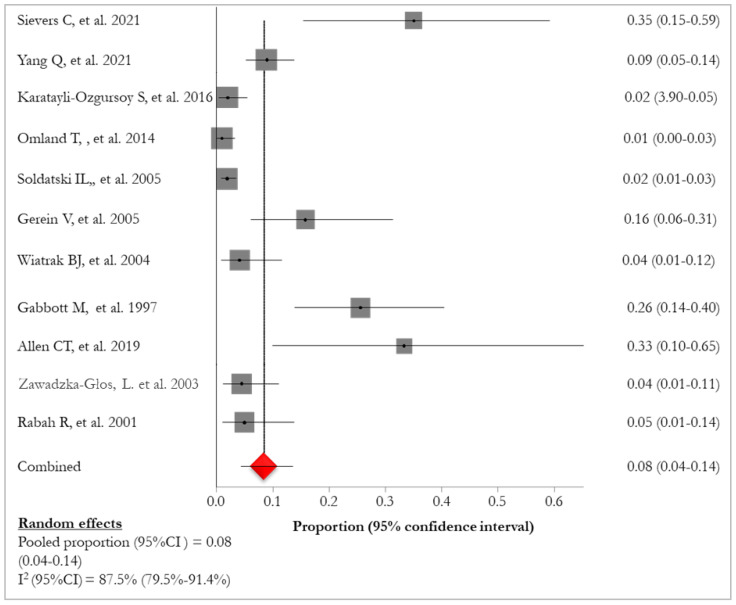
Forest plot of prevalence of lung involvement in RRP patients [25,26,27,28,29,30,31,32,33,34,35]. The red diamond represents the overall pooled effect from the included studies.

Lung involvement and/or pulmonary lesions were described with the following definitions: “lung/pulmonary involvement” [26,30,34], “pulmonary spread” [28], “pulmonary disease” [25,32,33], “pulmonary lesion” [31], “pulmonary papillomatosis” [27,29], and “development of dysplasia” [35]. The pooled prevalence was 8% (95% CI: 4–14%; I^2^: 87.5%), ranging from 1% [35] to 35% [25] (Table 4; Figure 2).

**Figure 3:**
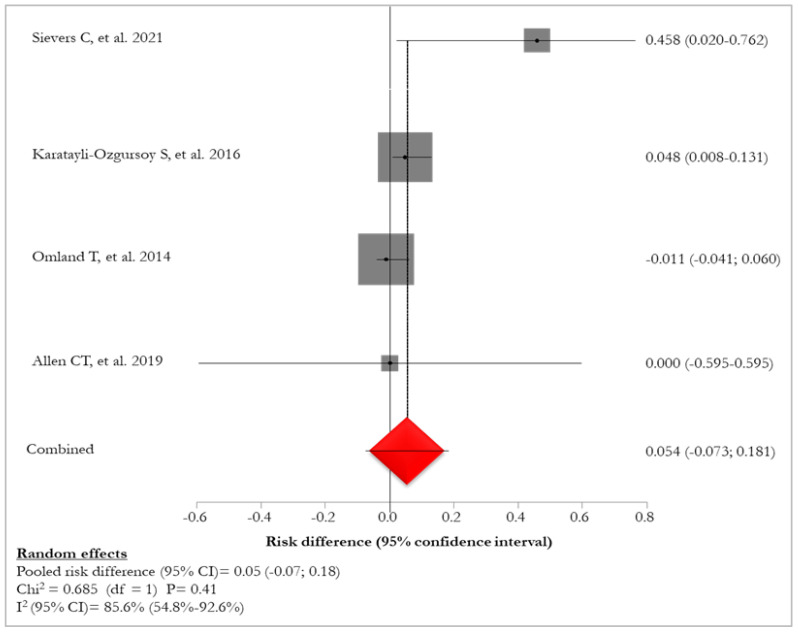
Forest plot of risk difference of pulmonary involvement between JORRP and AORRP patients [25,26,31,35]. The red diamond represents the overall pooled effect from the included studies.

Pooled risk difference of lung involvement between JORRP and AORRP was 5% (95% CI: −7–18%; I^2^: 85.6%), ranging from −1.1% [33] to 4.5% [26] (*p*-value: 0.41) (Figure 3) [27,32,34,35].

**Figure 4:**
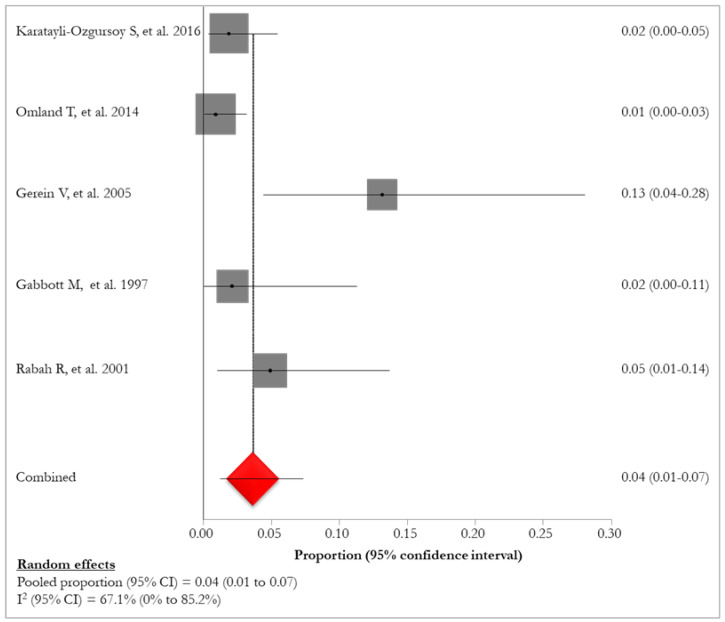
Forest plot of prevalence of lung tumor in RRP patients [26,28,30,33,35]. The red diamond represents the overall pooled effect from the included studies.

Lung tumor was reported in five (45.5%) studies [26,28,30,33,35] with a pooled prevalence of 4% (95% CI:1–7%; I^2^: 67.1%) ranging from 1% [35] to 13% [28] (Figure 3); the following definitions were used to assess the prevalence of lung tumor in RRP patients: “invasive pulmonary carcinoma ex-papillomatosis” [26], “lung carcinoma” [35], “pulmonary malignant transformation” [28], “pulmonary/lung squamous cell carcinoma” [30,33] (Table 5, Figure 4).

**Figure 5:**
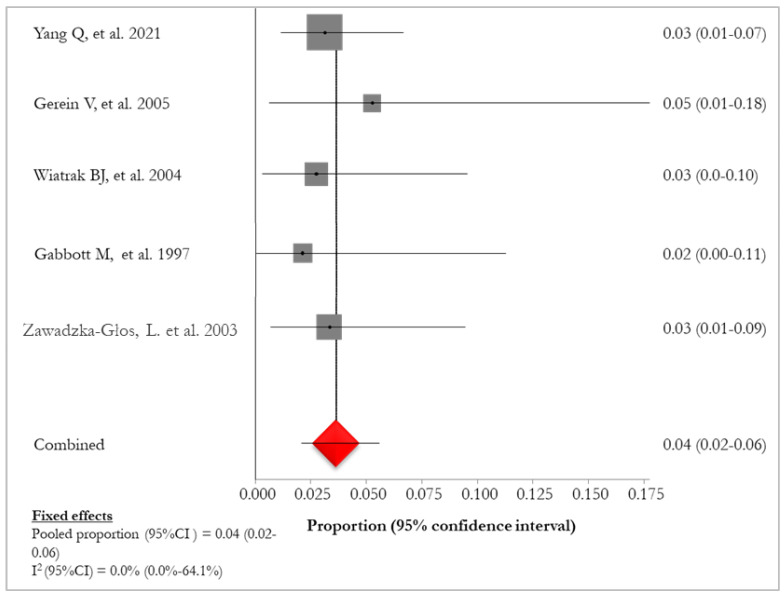
Forest plot of mortality in RRP [28,29,30,32,34]. The red diamond represents the overall pooled effect from the included studies.

The pooled mortality in patients with lung involvement (14/42, 33.3%) [28,29,30,32,34] was 4% (95% CI:2–6%; I^2^: 0%), ranging from 2% [30] to 5% [28] (Figure 5). Mortality of lung tumor (7/17, 41.2%) was reported in four (36.4%) studies [27,28,30,33] (Table 6).

#### Prevalence of HPV-6 and -11 Infections in Study Population

**Figure 6:**
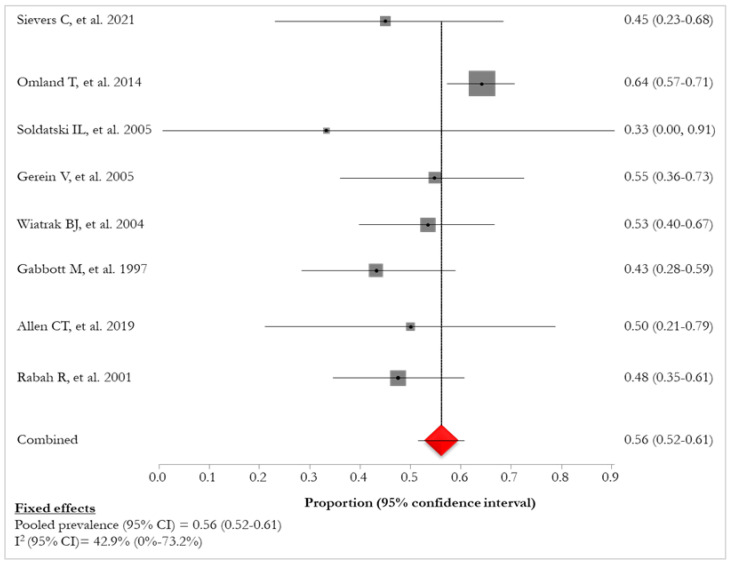
Prevalence of HPV-6 infections in patients with RRP [25,27,28,29,30,31,33,35]. The red diamond represents the overall pooled effect from the included studies.

Eight (72.7%) studies [25,27,28,29,30,31,33,35] reported a prevalence of HPV-6 or -11 of 91% (436/480). The pooled prevalence of HPV-6 was 56% (95% CI: 52–61%; I^2^: 42.9%), ranging from 33% [27] to 64% [35] (Figure 6, Table 7 and Table 8).

**Figure 7:**
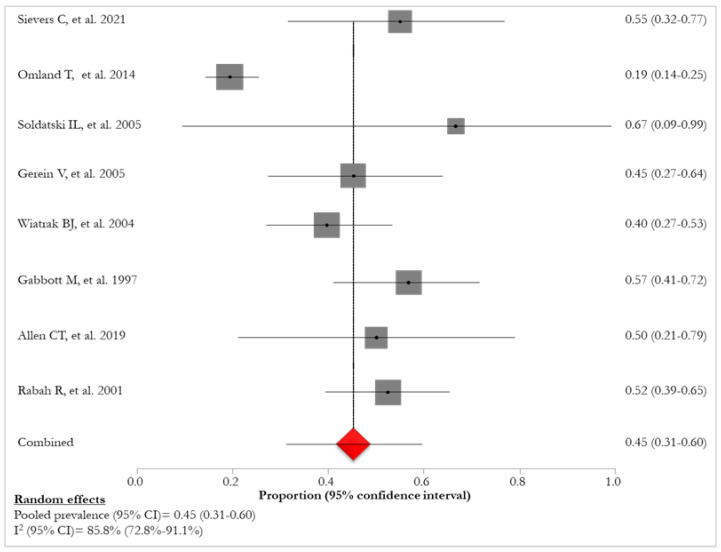
Prevalence of HPV-11 infections in patients with RRP [25,27,28,29,30,31,33,35]. The red diamond represents the overall pooled effect from the included studies.

The pooled prevalence of HPV-11, reported in eight studies [24,26,27,28,29,30,32,35], was 45% (95% CI:31–60%; I^2^:85.8%), varying from 19% [26] to 67% [35] (Figure 7). 

**Figure 8:**
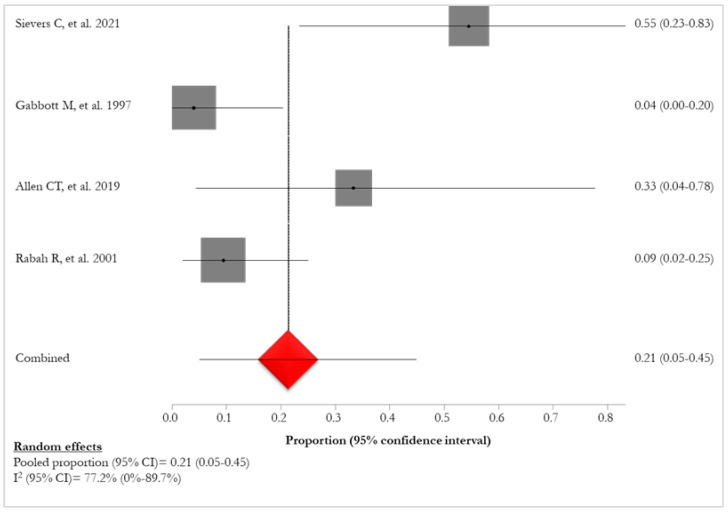
Forest plot of lung involvement in RRP HPV-11 positives [25,30,31,33]. The red diamond represents the overall pooled effect from the included studies.

A higher prevalence of HPV-11 (4/8, 50%) [24,29,30,32] was found in patients with lung involvement in comparison to those with HPV-6 infection (2/8, 25%) [24,30]. The pooled proportion of HPV-11 positivity of patients with lung involvement was 21% (95% CI: 5–45%; I^2^: 77.2%), ranging from 4% [29] to 55% [24] (Figure 8).

## 4. Discussion

Pulmonary involvement in patients with RRP can be a serious and potentially severe complication. The present systematic review, despite the high in-between studies heterogeneity, mainly associated with population and study design differences, showed a prevalence of lung involvement of 8%. The estimates are partially similar to what was reported in the scientific literature. Specifically, recent reviews focusing on individuals aged less than 20 years demonstrated an estimated incidence of lung involvement equal to 3.3% and an estimated incidence of lung tumor of 16% in the same category of patients [18]. In agreement with our results, Pai and Colleagues (2022) reported an incidence of 8.9% of pulmonary involvement in the RRP population, with a higher risk in younger patients, in those undergoing multiple surgical operations, in those tracheostomized, and in those with tracheal involvement [14].

However, the variability of study-related definitions of pulmonary involvement, such as lung/pulmonary involvement, pulmonary lesions, pulmonary spread, pulmonary disease, or pulmonary papillomatosis, referring to the development and progression of pulmonary parenchymal lesions of laryngotracheal papillomatosis, could favor epidemiological bias of the estimates. 

In addition, our findings highlight the absence of differences between juvenile- and adult-onset RRP, despite the estimated prevalence, which was found to be slightly high in young patients. Notably, more studies (6/11) described lung involvement or cases of lung cancer only in JORRP, with the juvenile population size being larger than that of the adult group (950 vs. 290, respectively). Juvenile RRP patients with lung involvement may evolve into having a more aggressive disease, needing more frequently surgical interventions, and being associated with lower treatment success rates [18,36]. Currently, the two most frequently prescribed drugs are Interferon and Cidofovir, although the evidence on their effectiveness in RRP patients with pulmonary involvement is poor because of the poor number of cases and the long-term follow-up needed to evaluate the recurrence rate [18]. In addition, alternative chemotherapy-free treatment for RRP cases with pulmonary involvement was evaluated. The addition of a neoantigen vaccine to bevacizumab and immune checkpoint inhibitors (ICIs) has shown efficacy in inhibiting tumor cell replication, ensuring higher specificity and reduced toxicity [37]. Recently, several clinical trials demonstrated the efficacy of investigational DNA immunotherapy (e.g., INO-3107 and PRGN-2012) for recurrence in AORRP, following the elicitation of T-cell responses versus E6/E7 oncoproteins of HPV-6 and -11 [38,39].

In agreement with previous studies, both the overall prevalence of lung tumors and mortality in JORRP and AORRP was 4% [20,40,41]. The development of lung involvement, as well as further progression to cancer, could be related to several factors, such as integration of HPV in host genome and host characteristics. However, specific studies focused on the pathogenesis of complications in RRP patients are currently scanty [42,43] and could partially hinder the assessment of new therapeutic options. 

Contrary to what was described in other HPV-related diseases, where the development of cancerous lesions is mainly driven by HR-HPV genotypes [44,45,46], a significant role played by low-risk HPV genotypes is confirmed, especially for HPV-6 and HPV-11 genotypes. In fact, despite their “low risk” classification, those genotypes can trigger cellular proliferation and transformation into dysplasia and carcinoma [47]. In total, 8/11 studies (72.7%) detected HPV genotypes, with a positivity of 91% for HPV-6 or -11: a pooled estimated prevalence of 56% and 45% were found for HPV-6 and HPV-11, respectively. Notably, considering only cases with lung involvement, the prevalence of HPV-11 infection was 21%, in agreement with previous studies, which demonstrated that infections caused by HPV-11 can cause more severe disease and an increased risk of cancer when compared with those caused by HPV-6 [41,48]. As highlighted in other HPV-related diseases in the recent past, our study highlights the importance of standardization of HPV testing and genotyping of RRP lesions. This could provide the background for further studies aimed at evaluating the mechanisms related to lung involvement, tumor development, and risk of malignant transformation [18]. Moreover, future research should be addressed to evaluate the risk of malignant transformation associated with different HPV genotypes.

Our review had several limitations, mainly related to the poor quality of the selected studies and to missing variables, which could affect the precision of the estimates. These features highlight the importance of conducting a more comprehensive analysis, implying more appropriate study designs. Further longitudinal studies could provide key information on progression from RRP to pulmonary diseases to support the development of new tools for diagnosis and treatment of infected patients.

Despite the limitations of the review, including the overall poor quality of some studies and missing variables, complexities and challenges associated with the study of pulmonary involvement in RRP should be emphasized, underscoring the need of further observational and experimental research to improve patient outcomes: studies tailored to those objectives could provide crucial insights on the progression from RRP to pulmonary diseases, favoring the identification and development of new diagnostic and treatment strategies for RRP patients.

## 5. Conclusions

According to the current evidence, a prevalence of lung involvement of 8% was found in RRP patients, without significant differences between adult and young patients. In comparison with other HPV-related diseases, a key role of low-risk HPV genotypes in RRP development and progression to severe diseases was described. In conclusion, lung involvement as well as progression to lung cancer in patients with RRP represent a clinical not negligible consequence, particularly in young patients who require frequent surgical interventions with associated low-success treatment rates. The findings of the present systematic review highlight the need for more in-depth studies to elucidate the mechanisms behind the occurrence of lung involvement and explore the risk factors associated with different HPV genotypes and their interaction with host immune responses in RRP patients. 

## Figures and Tables

**Table 1:** Checklist for cohort studies ^1^ according to the Scottish Intercollegiate Guidelines Network.

Ref.	Study	Q1	Q2	Q3	Q4	Q5	Score	Grade of Evidence ^2^
[25]	Sievers C, 2021	No	No	No	Yes	No	1	-
[34]	Yang Q, 2021	Yes	Yes	No	No	Yes	3	
[26]	Karatayli-Ozgursoy S, 2016	Yes	No	No	No	No	1	-
[35]	Omland T, 2014	Yes	Yes	No	No	Yes	3	
[27]	Soldatski IL, 2005	Yes	No	No	No	Yes	2	-
[28]	Gerein V, 2005	Yes	No	No	Yes	No	2	-
[29]	Wiatrak BJ, 2004	Yes	No	No	Yes	No	2	-
[30]	Gabbott M, 1997	Yes	No	No	Yes	No	2	-
[32]	Zawadzka-Głos L, 2003	Yes	No	No	Yes	No	2	-
[33]	R. Rabah, 2001	Yes	No	No	No	No	1	-

^1^ One score for each checkpoint: Q1: Are both groups selected from the same and well-defined cohort? Q2: Is the proportion of dropout in each group known, and if so, is it <15% in each? Q3: Any comparison between full participants and those lost to follow-up? Q4: Main potential confounders identified and considered? Q5: Any confidence interval? ^2^ Grading was refined with a ‘+’ sign to suggest a low risk of bias for a score of 4 or 5, a ‘–’ sign to suggest a high risk of bias for a score of 1 or 2, and no sign to suggest a moderate risk of bias for a score of 3. Scottish Intercollegiate Guidelines Network. SIGN 50: a guideline developer’s handbook. Edinburgh, UK: SIGN, 2014.

**Table 2:** JBI risk of bias assessment table. Nine items per study were evaluated, and the risk of bias was calculated on the number of positive answers. Y = yes, n = no, u = unclear. The risk of bias was evaluated as high, moderate, or low if the percentage of positive answers were, respectively, ≤49%, between 50% and 75%, or above 75%.

Ref.	First Author	Q1	Q2	Q3	Q4	Q5	Q6	Q7	Q8	Q9	Overall Appraisal
[31]	Allen C.T.; 2019	Yes	No	Not applicable	No	Yes	Yes	Not applicable	Yes	Unclear	High Risk

Q1: Is it clear in the study what is the “cause” and what is the ‘effect’ (i.e., there is no confusion about which variable comes first)? Q2: Were the participants included in any comparisons similar? Q3: Were the participants included in any comparisons receiving similar treatment/care, other than the exposure or intervention of interest? Q4: Was there a control group? Q5: Were there multiple measurements of the outcome both pre and post the intervention/exposure? Q6: Was follow-up complete, and if not, were differences between groups in terms of their follow-up adequately described and analyzed? Q7: Were the outcomes of participants included in any comparisons measured in the same way? Q8: Were outcomes measured in a reliable way? Q9: Was appropriate statistical analysis used?

**Table 3:** Summary of the main characteristic of the included studies.

Ref	First Author	Title	Publication Year	Type of Study	Mono/ Multicenter Study	Country	Study Period	Follow-Up
[25]	Sievers C, et al.	Comprehensive multiomic characterization of human papillomavirus-driven recurrent respiratory papillomatosis reveals distinct molecular subtypes	2021	-	-	USA	12 months	-
[34]	Yang Q, et al.	Long-term Outcomes of Juvenile Onset Recurrent Respiratory Papillomatosis with Pulmonary Involvement	2021	Retrospective-Observational	Monocenter	China	29 years (January 1990–October 2019)	10 years
[26]	Karatayli-Ozgursoy S, et al.	Risk Factors for Dysplasia in Recurrent Respiratory Papillomatosis in an Adult and Pediatric Population	2016	Retrospective	Monocenter	USA	8 years (July 2005–December 2013)	-
[35]	Omland T, et al.	Recurrent respiratory papillomatosis: HPV genotypes and risk of high-grade laryngeal neoplasia	2014	Retrospective	Multicenter	Norway	22 years (1987–2009)	Until 2012
[27]	Soldatski IL, et al.	Tracheal, bronchial, and pulmonary papillomatosis in children	2005	Retrospective	Monocenter	Russia	15 years (1988–2003)	-
[28]	Gerein V, et al.	Incidence, age at onset, and potential reasons of malignant transformation in recurrent respiratory papillomatosis patients: 20 years experience	2005	Prospective	Multicenter	Germany	7 years (1983–1990)	Until 2003
[29]	Wiatrak BJ, et al.	Recurrent respiratory papillomatosis: a longitudinal study comparing severity associated with human papilloma viral types 6 and 11 and other risk factors in a large pediatric population	2004	Prospective-Longitudinal	Monocenter	USA	10 years	-
[30]	Gabbott M, et al.	Human papillomavirus and host variables as predictors of clinical course in patients with juvenile-onset recurrent respiratory papillomatosis	1997	Retrospective	Monocenter	Australia	15 years (1981–1996)	-
[31]	Allen CT, et al.	Safety and clinical activity of PD-L1 blockade in patients with aggressive recurrent respiratory papillomatosis	2019	Interventional/phase II	Monocenter	USA	-	18 years
[32]	Zawadzka-Głos L, et al.	Lower airway papillomatosis in children	2003	Observational	Monocenter	Poland	22 years (1980–2002)	8–16 years
[33]	R. Rabah, et al.	Human papillomavirus-11-associated recurrent respiratory papillomatosis is more aggressive than human papillomavirus-6-associated disease	2001	Retrospective	Monocenter	USA	20 years (1979–1999)	-

**Table 4:** Prevalence of lung involvement in RRP patients [24,25,26,27,28,29,30,31,32,34,35].

Ref	First Author	N° Lung Involvement	% Lung Involvement
[25]	Sievers C., 2021	7/20	35.0
[34]	Yang Q., 2021	17/192	8.9
[26]	Karatayli-Ozgursoy S., 2016	3/159	1.9
[35]	Omland T., 2014	2/224	1.0
[27]	Soldatski IL., 2005	8/448	1.8
[28]	Gerein V., 2005	6/38	16.0
[29]	Wiatrak BJ., 2004	3/73	4.1
[30]	Gabbott M., 1997	12/47	26.0
[31]	Allen CT., 2019	4/12	33.3
[32]	Zawadzka-Głos L., 2003	4/90	4.4
[33]	R. Rabah., 2001	3/61	4.9

**Table 5:** Prevalence of lung tumor in RRP patients [26,28,30,33,35].

Ref	First Author	N° Lung Tumor	% Lung Tumor
[26]	Karatayli-Ozgursoy S., 2016	3/159	1.9
[35]	Omland T., 2014	2/224	0.9
[28]	Gerein V., 2005	5/38	13.1
[30]	Gabbott M., 1997	1/12	8.3
[33]	R. Rabah., 2001	3/61	4.9

**Table 6:** Frequencies and percentages of mortality in RRP patients [28,29,30,32,34].

Ref	First Author	Mortality (n)	Mortality (%)
[34]	Yang Q., 2021	6/17	35.3
[28]	Gerein V., 2005	2/6	33.3
[29]	Wiatrak BJ., 2004	2/3	66.6
[30]	Gabbott M., 1997	1/12	8.3
[32]	Zawadzka-Głos L., 2003	3/4	75.0

**Table 7:** Overall prevalence of HPV infection [25,27,28,29,30,31,33,35].

Ref	First Author	Sample Size for Analyses	HPV Positivity, n (%)
[25]	Sievers C., 2021	20	20 (100.0)
[35]	Omland T., 2014	221	207/221 (93.7)
[27]	Soldatski IL., 2005	8	3/8 (37.5)
[28]	Gerein V., 2005	38	31/38 (81.6)
[29]	Wiatrak BJ., 2004	73	58/73 (79.5)
[30]	Gabbott M., 1997	47	44/47 (93.6)
[31]	Allen CT., 2019	12	12 (100.0)
[33]	R. Rabah., 2001	61	61 (100.0)

**Table 8:** Prevalence of HPV-6 and HPV-11 infection.

Ref	First Author	Sample Size for Analyses	HPV-6 Positivity, n (%)	HPV-11 Positivity, n (%)
[25]	Sievers C., 2021	20	9/20 (45.0)	11/20 (55.0)
[35]	Omland T., 2014	221	133/207 (64.2)	40/207 (19.3)
[27]	Soldatski IL., 2005	8	1/3 (33.3)	2/3 (66.7)
[28]	Gerein V., 2005	38	17/38 (44.7)	14/38 (36.8)
[29]	Wiatrak BJ., 2004	73	31/58 (53.5)	23/58 (39.7)
[30]	Gabbott M., 1997	47	19/44 (43.2)	25/45 (56.8)
[31]	Allen CT., 2019	12	6/12 (50.0)	6/12 (50.0)
[33]	R. Rabah., 2001	61	29/61 (47.5)	32/61 (52.5)

## Data Availability

The data are available in case it is requested for motivated reasons.

## Supplementary Materials

The following supporting information can be downloaded at: https://www.mdpi.com/article/10.3390/idr16020016/s1. Table S1. Summary of the main characteristic of the included studies.
