# Supplementary material for: Pulmonary Involvement in Recurrent Respiratory Papillomatosis: A Systematic Review
Source: Infect Dis Rep. 2024 Feb 28;16(2):200–15. doi: 10.3390/idr16020016 (PMC10961772; doi:10.3390/idr16020016)
Supplement: Supplementary file 1 [file idr-16-00016-s001.zip › idr-2825224-supplementary.pdf]

S1. Table S1. Summary of the main characteristic of the included studies.

Ref.	First Author	Title	Publication year	Type of study	Mono/Multicenter Study	Country	Study period	Follow-up
[24]	Sievers C, et al.	Comprehensive multiomic characterization of human papillomavirus-driven recurrent respiratory papillomatosis reveals distinct molecular subtypes	2021	-	-	USA	12 months	-
[25]	Yang Q, et al.	Long-term Outcomes of Juvenile Onset Recurrent Respiratory Papillomatosis with Pulmonary Involvement	2021	Retrospective-Observational	Monocenter	China	29 years (January 1990-October 2019)	10 years
[34]	Karatayli-Ozgursoy S, et al.	Risk Factors for Dysplasia in Recurrent Respiratory Papillomatosis in an Adult and Pediatric Population	2016	Retrospective	Monocenter	USA	8 years (July 2005-December 2013)	-
[26]	Omland T, et al.	Recurrent respiratory papillomatosis: HPV genotypes and risk of high-grade laryngeal neoplasia	2014	Retrospective	Multicenter	Norway	22 years (1987-2009)	Until 2012
[35]	Soldatski IL, et al.	Tracheal, bronchial, and pulmonary papillomatosis in children	2005	Retrospective	Monocenter	Russia	15 years (1988-2003)	-
[27]	Gerein V, et al.	Incidence, age at onset, and potential reasons of malignant transformation in recurrent respiratory papillomatosis patients: 20 years experience	2005	Prospective	Multicenter	Germany	7 years (1983-1990)	Until 2003

[28]	Wiatrak BJ, et al.	Recurrent respiratory papillomatosis: a longitudinal study comparing severity associated with human papilloma viral types 6 and 11 and other risk factors in a large pediatric population	2004	Prospective-Longitudinal	Monocenter	USA	10 years	-
[29]	Gabbott M, et al.	Human papillomavirus and host variables as predictors of clinical course in patients with juvenile-onset recurrent respiratory papillomatosis	1997	Retrospective	Monocenter	Australia	15 years (1981-1996)	-
[30]	Allen CT, et al.	Safety and clinical activity of PD-L1 blockade in patients with aggressive recurrent respiratory papillomatosis	2019	Interventional/phase II	Monocenter	USA	-	18 years
[31]	Zawadzka-Glos L, et al.	Lower airway papillomatosis in children	2003	Observational	Monocenter	Poland	22 years (1980-2002)	8-16 years
[32]	R. Rabah, et al.	Human papillomavirus-11-associated recurrent respiratory papillomatosis is more aggressive than human papillomavirus-6-associated disease	2001	Retrospective	Monocenter	USA	20 years (1979-1999)	-
